# *Chlamydia trachomatis* Serovars Drive Differential Production of Proinflammatory Cytokines and Chemokines Depending on the Type of Cell Infected

**DOI:** 10.3389/fcimb.2019.00399

**Published:** 2019-11-26

**Authors:** Robert Faris, Shelby E. Andersen, Alix McCullough, Françoise Gourronc, Aloysius J. Klingelhutz, Mary M. Weber

**Affiliations:** Department of Microbiology and Immunology, University of Iowa Carver College of Medicine, Iowa City, IA, United States

**Keywords:** *Chlamydia trachomatis*, innate immune response, trachoma, serovariant, macrophage

## Abstract

*Chlamydia trachomatis* serovars A-C infect conjunctival epithelial cells and untreated infection can lead to blindness. D-K infect genital tract epithelial cells resulting in pelvic inflammatory disease, ectopic pregnancy, and sterility while L1-L3 infect epithelial cells and macrophages, causing an invasive infection. Despite some strains of *Chlamydia* sharing high nucleotide sequence similarity, the bacterial and host factors that govern tissue and cellular tropism remain largely unknown. Following introduction of *C. trachomatis* via intercourse, epithelial cells of the vagina, foreskin, and ectocervix are exposed to large numbers of the pathogen, yet their response to infection and the dynamics of chlamydial growth in these cells has not been well-characterized compared to growth in more permissive cell types that harbor *C. trachomatis*. We compared intracellular replication and inclusion development of representative *C. trachomatis* serovars in immortalized human conjunctival epithelial, urogenital epithelial, PMA stimulated THP-1 (macrophages), and HeLa cells. We demonstrate that urogenital epithelial cells of the vagina, ectocervix, and foreskin restrict replication of serovar A while promoting robust replication and inclusion development of serovar D and L2. Macrophages restrict serovars D and A while L2 proliferates in these cells. Furthermore, we show that GM-CSF, RANTES, GROα, IL-1α, IL-1β, IP-10, IL-8, and IL-18 are produced in a cell-type and serovar-specific manner. Collectively we have established a series of human cell lines that represent some of the first cell types to encounter *C. trachomatis* following exposure and show that differential production of key cytokines early during infection could regulate serovar-host cell specificity.

## Introduction

*Chlamydia trachomatis* is an important human pathogen and the cause of blinding trachoma and a sexually transmitted infection. *C. trachomatis* isolates exist as 15 serovariants that are subdivided into two major biovars: trachoma, which consists of ocular tropic strains (A, B, Ba, and C) and genitourinary tract tropic (D, E, F, G, H, I, J, and K) strains, and invasive lymphogranuloma venereum (L1, L2, and L3) (Elwell et al., 2016). Infections of the lower genital tract are often asymptomatic and thus may go untreated, leading to ascending infections. In women, ascending infections can result in severe life-long complications such as pelvic inflammatory disease (PID), ectopic pregnancy, sterility, and chronic pelvic pain (Darville and Hiltke, 2010; Malhotra et al., 2013) while in men, infection can lead to urethritis, balantitis, and can ascend resulting in epididymitis, swelling of the seminal vesicles, and potentially prostatitis (Nickel, 2003; Redgrove and Mclaughlin, 2014). Men with an uncircumcised penis are significantly more likely to transmit *C. trachomatis* to their female partners during vaginal intercourse (Castellsague et al., 2005; Turner et al., 2010) however, it is unknown as to whether *C. trachomatis* can actually infect the human foreskin. LGV strains can infect mucosal surfaces but uniquely infect macrophages, causing an invasive sexually transmitted infection that disseminates to the regional draining lymph nodes (Lausen et al., 2018), although the adaptations that allow these strains to survive and proliferate in macrophages are mostly unknown. Infection with LGV isolates can cause genital ulcers, lymphadenopathy (buboes), fibrosis, and fistulae; ultimately resulting in damage to the mucosal epithelium and scarring (Rawla and Limaiem, 2019). Serovars A-C infect conjunctival epithelial cells and untreated infection results in entropion, trichiasis, opacification, and blindness (Hu et al., 2013). While all chlamydial isolates are able to infect the conjunctiva mucosa, only infection with ocular tropic trachoma isolates can cause blindness whereas infection with urogenital isolates results in conjunctivitis that is usually self-limiting (Hu et al., 2013). Furthermore, most ocular tropic trachoma isolates rarely infect the genital mucosa (Hu et al., 2013). Thus, there is a specificity of different serovars for different cell types.

All chlamydiae exhibit a biphasic developmental cycle in which the bacteria alternate between two forms: an infectious elementary body (EB) and the replicative reticulate body (RB) (Abdelrahman and Belland, 2005). During infection, the EB is internalized into a membrane-bound compartment that is extensively modified by the pathogen to establish its replicative niche termed the inclusion (Scidmore-Carlson et al., 1999; Weber et al., 2015). The inclusion avoids fusion with lysosomes and traffics along microtubules to the peri-Golgi region (Grieshaber et al., 2003; Scidmore et al., 2003). Throughout the infection cycle, the inclusion interacts with select host organelles to acquire key nutrients, including lipids, amino acids, and iron, while avoiding activation of innate immune defenses (Elwell et al., 2016). At the end of the developmental cycle, EBs are released from the host cell by cell lysis or extrusion (Hybiske and Stephens, 2007), allowing the infection cycle to begin anew.

Ocular, genital, and LGV serovars can infect a plethora of cell-types and tissues however; the pathology associated with infection, tissue tropism, and the clinical outcome of infection is serovar and tissue specific. For instance, serovar D infection of the conjunctival epithelium can cause conjunctivitis while serovar A infection can cause blinding trachoma (Hu et al., 2013). Likewise, serovar A infection of the genitourinary tract usually does not lead to a productive infection. According to the cellular model of chlamydial pathogenesis, pathology associated with *Chlamydia* infection is a consequence of the cellular response initiated by infected non-immune cells (Stephens, 2003). Infection of epithelial cells, which are the primary targets of *Chlamydia*, results in production of cytokines and chemokines such as IL-8, IL-6, and GM-CSF that recruit inflammatory leukocytes to the site of infection (Rasmussen et al., 1997; Dessus-Babus et al., 2002). Neutrophils, macrophages, B-cells, and T-cells release their own repertoire of inflammatory mediators that contribute to the inflammatory response, but also damage the epithelium (Ramsey and Rank, 1991; Morrison et al., 1995; Stephens, 2003). The release of growth factors and proteases from infected cells further contributes to tissue remodeling, tissue damage, and scarring (Rasmussen et al., 1997). Understanding how the host responds to *C. trachomatis* in specific cell-types and tissues is essential to understanding the pathological consequences of chlamydial infection.

Despite its significant impact on global human health, how and why serovariants infect specific cell types, different tissues, and cause distinct pathology remains largely unknown. The majority of studies evaluating the host innate immune response to *C. trachomatis* have focused on a single serovar and a single cell-type (Miyairi et al., 2006; Buckner et al., 2013; Cunningham et al., 2013; Giakoumelou et al., 2017). Most studies have used human cervical carcinoma epithelioid (HeLa), human laryngeal carcinoma (Hep2), or murine fibroblast cells to model *Chlamydia* infection (Rasmussen et al., 1997; Dessus-Babus et al., 2002; Cunningham et al., 2013) while others have focused on epithelial cells of the endocervix (Ibana et al., 2012; Buckner et al., 2013). While *Chlamydia* can infect and replicate in a plethora of cell types, recent studies have shown that cells derived from different anatomical sites support different levels of *Chlamydia* replication and inclusion development (Miyairi et al., 2006; Jolly et al., 2019). Furthermore, HeLa cells, the most common model for studying chlamydial infection, are known to have many abnormalities and the immune signaling pathways of these cells have been altered over the years through high passage *in vitro* growth (Masters, 2002; Landry et al., 2013). Although some studies have evaluated aspects of chlamydial infection and host response in cell-types known to harbor the pathogen in women (e.g., endocervical cells) (Lewis et al., 2014), these cells are more permissive to chlamydial growth and are likely infected following ascension of the pathogen and are ideal models to study later stages of *Chlamydia* infection but not the best models to understand early events and potential barriers to serovar-specific infection. Ectocervical cells likely encounter *Chlamydia* prior to endocervical cells and thus, we postulate that these cells play a major “gate-keeping” function in preventing ascension of the pathogen. We speculate that ectocervical cells support chlamydial growth but also limit chlamydial infection and determine serovar specificity via the release of immune modulators. Although endocervical cells are more permissive to chlamydial growth, ectocervical cells are indeed infected at approximately one third the rate, suggesting that these cells may determine whether *Chlamydia* infects the endocervix (Moorman et al., 1986).

Healthy, proliferating, estrogen fed cells of the stratified vaginal epithelium and ectocervical keratinocytes are likely some of the first cells to be exposed to and infected by *C. trachomatis* in women, making them critical players in determining whether the pathogen gains a foothold in the human body. Terminally differentiated superficial epithelial cells are usually metabolically inactive as they lose their mitochondria and lack nuclei, making them poor hosts for *C. trachomatis*. The human vaginal *Stratum Corneum* combined with the squamous epithelium forms a strong barrier against infection while other tissues like the columnar epithelium of the endocervix are easily infected, thus making these tissues much more prone to harbor and support chlamydial proliferation (Anderson et al., 2014). A recent study utilizing *in vitro* 3D organotypic systems has demonstrated that the differentiation state of stratified squamous epithelium determines these cells ability to support proliferation of *C. trachomatis* serovars D and L2 with undifferentiated cells being the most permissive to growth of both serovars (Nogueira et al., 2017). Thus, some chlamydial serovars are able to infect various cell types of the stratified vaginal epithelium and these cells likely determine the tempo of chlamydial infection. However, the immune response by the vaginal epithelium in response to serovar specific chlamydial infection is unknown.

In the current study, we employ very early passage immortalized primary human vaginal epithelial cells (HVEC) (Peterson et al., 2005), human ectocervical keratinocytes (HCK) (Sprague et al., 2002), human foreskin keratinocytes (HFK-2) (Kiyono et al., 1998), human conjunctival epithelial cells (HCjE) (Gipson et al., 2003; Derrick et al., 2016), and PMA stimulated THP-1 cells to evaluate invasion, intracellular replication, and the host response to trachoma (A/HAR-13), genital (D/UW-3/CX), and lymphogranuloma venereum (434/Bu) isolates. Our data shows that these urogenital cells restrict intracellular replication and inclusion development of serovar A, a phenomenon not observed in HeLa cells. Serovar D and L2 were able to infect, form normal inclusions in, and robustly proliferate in human foreskin keratinocytes demonstrating that these cells can indeed support chlamydial growth *in vitro* and suggesting that they may play a role early during infection of men. We further show that serovar L2 replicates in PMA stimulated THP-1 cells while replication of serovars A and D are significantly hindered. Cytokine profiling of L2, D, and A infected cells revealed that the host cell responds to *C. trachomatis* in a cell-type and serovar-specific manner. Notably, we show that GM-CSF, RANTES, GROα, IL-1α, IL-1β, IP-10, IL-8, and IL-18 are differentially produced in response to specific serovariants infecting specific cell-types. Furthermore, these data suggest that L2 may modulate cytokine production in epithelial cells to promote infiltration of the lymph.

## Materials and Methods

### Bacterial and Cell Culture

*Chlamydia trachomatis* 434/Bu, D/UW-3/CX, and A/HAR-13 were propagated in HeLa 229 cells (American Type Culture Collection) and infectious EBs were purified using a renografin density gradient as previously described (Caldwell et al., 1981). Work with all chlamydial strains was conducted in a BSL2 in accordance with guidelines set by the University of Iowa biosafety committee.

HeLa 229 (ATCC) and THP-1 cells (MilliporeSigma) were propagated in RPMI 1640 with L-glutamine (ThermoFisher) and supplemented with 10% Fetal Bovine Serum (VWR), 1 mM sodium pyruvate, and gentamicin. THP-1 cells were differentiated into macrophages using 100 nM phorbol myristate acetate (PMA) as described (Starr et al., 2018). The generation of the conjunctival and urogenital cell lines used in this study has been previously described (Sprague et al., 2002; Gipson et al., 2003) Immortalized human conjunctival epithelial cells (HCjE) (Gipson et al., 2003; Derrick et al., 2016), human vaginal epithelial cells (HVEC) (Peterson et al., 2005), human foreskin keratinocytes (HFK-2) (Kiyono et al., 1998), and human ectocervical keratinocytes (HCK) (Sprague et al., 2002) were cultured in keratinocyte serum free media (K-SFM) (ThermoFisher) supplemented with 0.16 ng/ml epidermal growth factor (EGF), 25 μg/ml bovine pituitary extract (BPE), and gentamicin. All cell lines were maintained at 37°C with 5% CO_2_. The purity and identity of the HCjE, HVEC, HFK-2, and HCK cells was confirmed through human STR profiling (ATCC) and the STR loci were analyzed using CLASTR1.4.3.

### Chlamydia Growth Curve

HeLa, HCjE, HVEC, HFK-2, and HCK were seeded at 10^5^/ml into 24-well plates. THP-1 cells were seeded into 24-well plates at 10^5^/ml and were differentiated into macrophages using 100 nM phorbol myristate acetate (PMA). Cells were infected in triplicate at a multiplicity of infection (MOI) of 1 with *C. trachomatis* 434/Bu, D/UW-3/CX, or A/HAR-13 using complete RPMI 1640 media. The inoculum was spun onto the cells at 900 × g for 15 min. Following centrifugation, the supernatant was removed and fresh RPMI 1640 media was added. Infected cells were incubated at 37°C with 5% CO_2_ for 0, 24, 48, or 72 h at which point host cells were lysed in water and serially diluted supernatants were applied to fresh HeLa cell monolayers. Bacterial titer plates were fixed in methanol and bacteria were stained with 1:1000 anti-LPS (Novus). Ten fields from triplicate samples were enumerated to determine the number of infectious forming units (IFUs). Data are representative of three independent experiments.

### Immunofluorescence Microscopy

HeLa, HCjE, HVEC, HFK-2, and HCK were seeded at 10^5^/ml onto glass coverslips in 24-well plates. THP-1 cells were seeded into 24-well plates at 10^5^/ml and were differentiated into macrophages using 100 nM PMA. Cells were infected in triplicate at a MOI of 1 with *C. trachomatis* 434/Bu, D/UW-3/CX, or A/HAR-13 using complete RPMI 1640 media. The inoculum was spun onto the cells at 900 × g for 15 min. Following centrifugation, the supernatant was removed and fresh RPMI 1640 media was added. Infected cells were incubated at 37°C with 5% CO_2_ for 24, 48, or 72 h. Cells were fixed in methanol and bacteria were stained with 1:1000 anti-LPS (Novus) and the inclusion membrane was visualized using 1:500 anti-IncE. Images were captured using a Nikon Eclipse Ti2 fluorescent microscope and analyzed using Nikon Elements software. Data are representative of 3 independent experiments with at least 100 infected cells per sample. To measure inclusion size, the area of 15 inclusions was measured in Image J using the LPS staining as the outline.

### Uptake Assay

HeLa, HCjE, HVEC, HFK-2, and HCK were seeded at 10^5^/ml onto glass coverslips in 24-well plates. THP-1 cells were seeded into 24-well plates at 10^5^/ml into 24-well plates and were differentiated into macrophages using 100 nM PMA. Cells were infected in triplicate at a MOI of 1 with *C. trachomatis* 434/Bu, D/UW-3/CX, or A/HAR-13 using complete RPMI 1640 media. The inoculum was spun onto the cells at 900 × g for 15 min. Following centrifugation, the supernatant was removed, cells where washed three times with RPMI and fresh RPMI media was added. Infected cells were incubated at 37°C with 5% CO_2_ for 30 min to stimulate uptake. Cells were fixed with 4% formaldehyde, blocked with 1% BSA, and bacteria were stained with 1:1000 anti-LPS (Novus) and anti-mouse DyLight 488 (ThermoFisher). Cells were subsequently permeabilized with 0.1% Triton-X 100 and bacteria were stained with 1:1000 anti-LPS (Novus) and anti-mouse DyLight 594 (ThermoFisher). DNA was stained with 1:1000 DAPI (ThermoFisher). Images were captured using a Nikon Eclipse Ti2 fluorescent microscope and analyzed using Nikon Elements software. Data are representative of 2 independent experiments with at least 20 fields imaged per experiment.

### Cytokine Array

HeLa, HCjE, HVEC, HFK-2, and HCK were seeded at 10^6^/ml into 6-well plates. THP-1 cells were seeded into 6-well plates at 10^6^/ml into 6-well plates and were differentiated into macrophages using 100 nM PMA. Cells were infected at a MOI of 1 with *C. trachomatis* 434/Bu, D/UW-3/CX, or A/HAR-13 using complete RPMI 1640 media. The inoculum was spun onto the cells at 900 x *g* for 15 min. Following centrifugation, the supernatant was removed and replaced with fresh RPMI media. When required, 50 μg/ml of chloramphenicol was added at 1 h post-infection. Infected cells were incubated at 37°C with 5% CO_2_ for 24 or 48 h. Supernatants and lysates were collected from each cell type and combined as many cytokines are rapidly secreted. Samples were assayed in duplicate for expression of 36 cytokines using the human cytokine array proteome profiler array following the manufacturers guidelines (R&D systems ARY005B). The cytokine array proteome profiler utilizes a dot blot with capture antibody pre-conjugated to the membrane for relative measurement of cytokines. To control for non-specific binding, each array contained both positive and negative controls. After the experimental procedure, array dot blots were exposed to X-ray film and developed. Image J was used to quantitate spot intensity for arrays. Measurements were acquired by auto-correcting for background based on the negative control spot and measuring the integrated density of each spot. For each array, a circle corresponding to the approximate size of the largest spot on the array (usually the reference spot) was made. This same circle was used to measure every spot on the blot without varying the size. Each array came with internal references spotted at different locations on the blot and present in sextuplet. For each array, the average integrated density of the reference was calculated as well as integrated densities for each spot on the array corresponding to the various cytokines. Each set of samples was averaged and a standard deviation between the duplicate samples was derived. The quotient of the integrated reference density and the cytokine specific spot density was used to represent the relative percentage of the specific spot density as a percentage of the reference density. This allowed control for slight variations in exposure time for different array dot blots. Although uncommon, some spots showed greater integrated density than standards, resulting in values in excess of 100%.

### Statistically Analysis

When appropriate, statistical significance was determined using One-Way ANOVA with Tukey as a post-test and yielded a significant difference of ^***^p < 0.001, ^**^p < 0.01, or ^*^p < 0.05. All statistically analysis was conducted using Prism software.

## Results

### Growth Comparison of *C. trachomatis* Serovars in Urogenital, Conjunctival, HeLa, and PMA Stimulated THP-1 Cells

**Figure 1:**
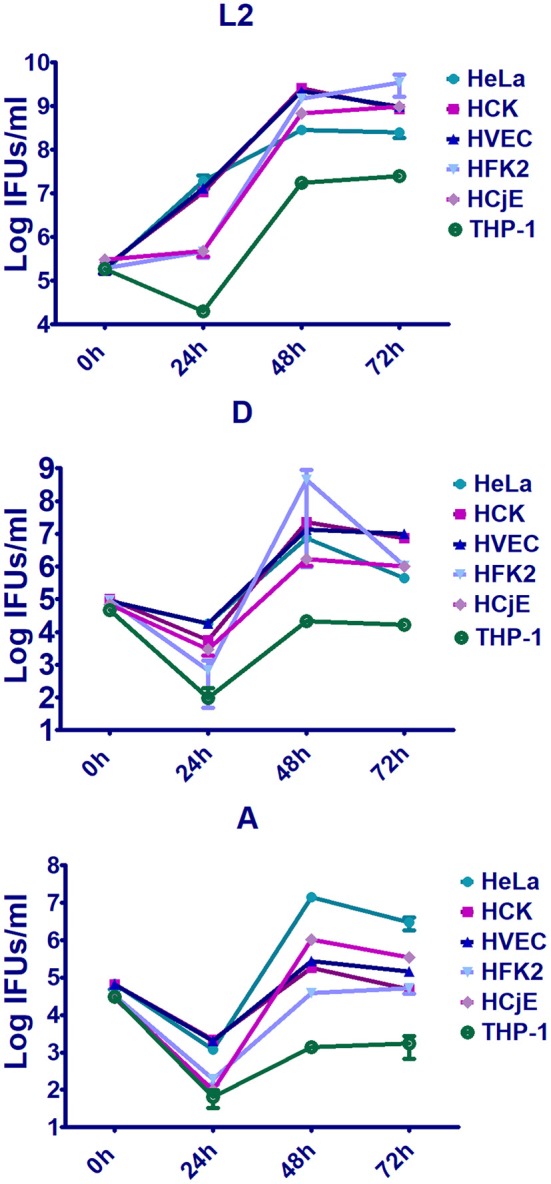
Replication of trachoma, urogenital, or LGV isolates in early passage immortalized urogenital cells, conjunctiva cells, PMA stimulated THP-1 cells, and HeLa cells. Human vaginal epithelial cells (HVEC), human foreskin keratinocytes (HFK-2), human ectocervical keratinocytes (HCK), human conjunctival epithelial cells (HCjE), PMA stimulated THP-1 cells, or HeLa cells were infected at a MOI of 1 with *C. trachomatis* 434/Bu (L2), D/UW-3/CX (D), and A/HAR-13 (A). At 0, 24, 48, or 72 h post-infection, host cells were lysed in water and lysates were plated on fresh HeLa monolayers to enumerate the number of infectious forming units (IFUs). Data are representative of 3 independent experiments.

Despite sharing high genomic sequence similarity and exhibiting nearly identical genomic synteny, *C. trachomatis* serovars preferentially infect distinct cell-types and the outcome and severity of disease depends on tissue and host susceptibility factors. To date, the reasons for these drastic differences remain largely unknown due to a lack of appropriate model systems and the fact that most studies have focused on a single serovar in a single cell-type. Here we compared bacterial intracellular replication of representative *C. trachomatis* serovars 434/Bu (L2), D/UW-3/CX (D), and A/HAR-13 (A) in human conjunctival epithelial cells (HCjE), human vaginal epithelial cells (HVEC), human foreskin keratinocytes (HFK-2), and human cervical keratinocytes (HCK) as well as in PMA stimulated THP-1 cells and HeLa cells. Enumeration of infectious forming units (IFUs) at 0, 24, 48, and 72 h revealed that urogenital cells (HVEC, HCK, and HFK-2) supported robust replication of serovars D and L2 (Figure 1, Figure S1), although serovar D replication was notably lower than that of L2 in all cell types. Strikingly, we observed a 1-2 log increase in bacterial burden compared to growth obtained using HeLa cells (Figure 1, Figure S1). In contrast to the urogenital isolates, we observed a 1-3 log decrease in growth of the trachoma isolate serovar A compared to that observed in HeLa cells (Figure 1, Figure S1). These results suggest that early passage immortalized urogenital cells promote robust replication of urogenital and LGV isolates while restricting trachoma isolates, a phenomenon not noted in HeLa cells. Collectively, our results show that these cells can support chlamydial growth in a serovar dependent manner and may be ideal models for exploring early events during infection.

To compare replication of the serovars in conjunctival epithelial cells, we infected HCjE cells with serovars L2, D, and A. Replication of each serovar was noted in these cells (Figure 1, Figure S1) although replication of serovars D and A were notably lower than L2. These data are in line with observations that all serovariants are able to infect the conjunctival epithelium.

To evaluate the ability of the serovariants to infect macrophages, THP-1 cells were treated with PMA followed by infection with *C. trachomatis* serovars L2, D, or A. While serovar L2 is able to infect and replicate in macrophages, all be it to a lower extend in epithelial cells, minimal replication of serovars D and A was noted (Figure 1, Figure S1). These results support observations that LGV is able to infect and replicate in macrophages whereas trachoma and urogenital isolates are unable to robustly proliferate in macrophages.

### Inclusion Development of *C. trachomatis* Serovars in Urogenital, Conjunctival, HeLa, and PMA Stimulated THP-1 Cells

**Figure 2:**
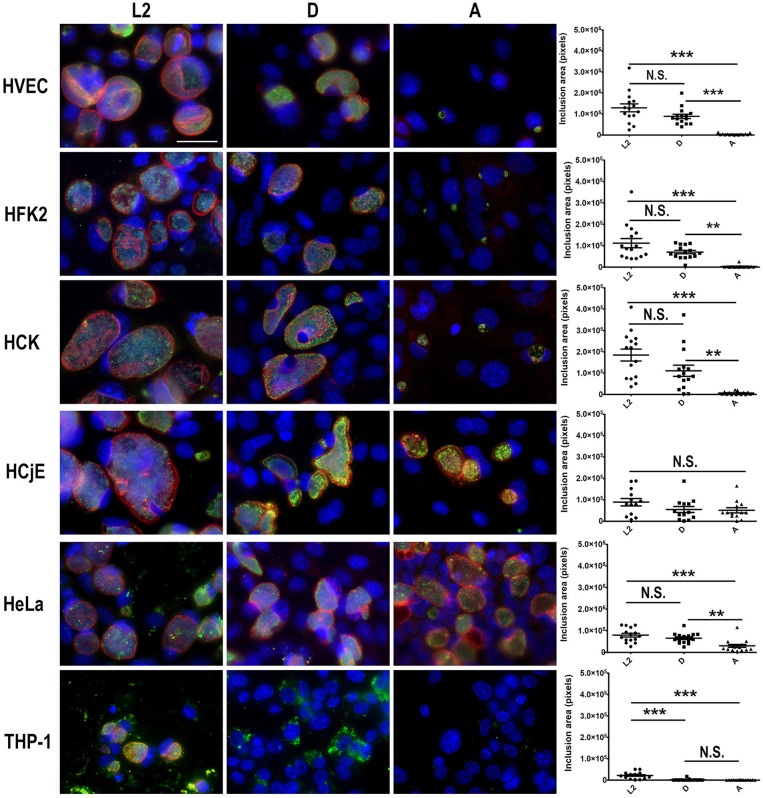
Comparison of inclusion development by trachoma, urogenital, or LGV isolates in urogenital, conjunctival epithelial cells, PMA stimulated THP-1 cells, and HeLa cells. HVEC, HFK-2, HCK, HCjE, or HeLa cells were infected at a MOI of 1 with *C. trachomatis* 434/Bu (L2), D/UW-3/CX (D), and A/HAR-13 (A). At 48 h post-infection, cells were fixed in methanol and stained using anti-LPS (green) to visualize the bacteria, anti-IncE (red) to illuminate the inclusion membrane, and DAPI (blue) to stain the host and bacterial DNA. Data are representative of 3 independent experiments. Scale bars are 50 nm. The inclusion area (pixels) was measured from 15 infected cells using Fiji. Statistical significance compared to uninfected cells (UI) was determined using One-way ANOVA with Tukey as a post-test and yielded a significant difference of ^***^p < 0.001 or ^**^p < 0.01.

Our results suggest that urogenital epithelial cells restrict replication of ocular tropic trachoma isolates and promote intracellular replication of urogenital and LGV isolates. Conversely, macrophages restrict serovar D and A replication but are permissive of L2 replication. To evaluate inclusion formation, each cell-type was infected with L2, D, or A and at 48 h post-infection cells were fixed and bacteria were stained using anti-LPS and the inclusion membrane was illuminated using an antibody specific for the inclusion membrane protein IncE. As shown in Figure 2, HVEC, HCK, and HFK-2 cells supported the formation of large, spacious L2 and D inclusions. Notably the inclusions were larger in HVEC, HCK, and HFK-2 than those obtained in HeLa cells (Figure 2). The presence of larger inclusions in these cells is in line with our observation that they support a ~1–2 log increase in bacterial burden compared to HeLa cells (Figure 1). Comparison of inclusions formed by serovar A in these cells revealed that the inclusions were significantly smaller than those formed by L2 or D and were strikingly smaller than those observed in HeLa cells (Figure 2). Uniquely in HeLa cells we occasionally observed the formation of aberrant serovar A inclusions in which only a few bacteria were present, but the inclusion appeared spacious as evident by IncE staining. Spacious inclusions were formed by serovars L2, D, and A in HCjE cells. Strikingly, for all serovars tested in HCjE cells, a gap between the IncE marker and the bacteria was observed, which was notably absent in all other cell types which showed tight IncE staining adjacent to the bacteria. Collectively these data support the results from our growth curves in which the urogenital epithelial cells: HVEC, HCK, and HFK-2, restrict replication of ocular tropic trachoma isolates whereas HCjE promote replication of all 3 isolates.

To evaluate inclusion development of serovars L2, D, and A in macrophages, we infected PMA stimulated THP-1 cells. As shown in Figure 2, inclusion development by serovar A was severely impaired in macrophages and only a few individual bacteria were observed 48 h post-infection. In contrast to serovar A, more serovar D bacteria were present; however, inclusion formation was still impaired. The findings for serovar D and A are in stark contrast to L2 in which inclusion formation was observed (Figure 2). While serovar L2 was able to replicate and form inclusions in macrophages, they were notably smaller than those observed in epithelial cells (Figure 2). Collectively the lack of inclusion formation by serovars A and D support our growth curve studies in which we show minimal increases in bacterial burden in macrophages.

### Differential Uptake of *C. trachomatis* Serovars by Urogenital, Conjunctival Cells, HeLa, and PMA Stimulated THP-1 Cells

**Figure 3:**
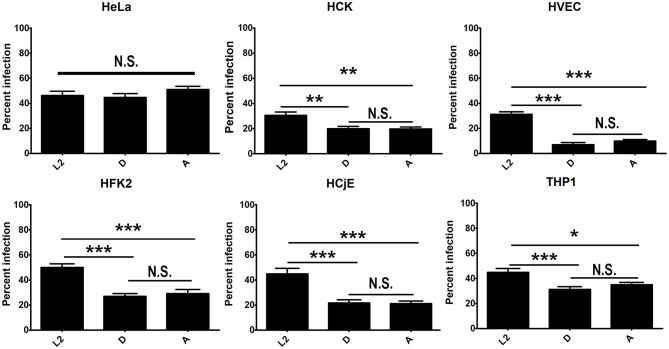
Differential uptake of *C. trachomatis* serovars by primary urogenital and conjunctival epithelial cells. HVEC, HFK-2, HCK, HCjE, or HeLa cells were infected at a MOI of 1 with *C. trachomatis* 434/Bu (L2), D/UW-3/CX (D), and A/HAR-13 (A). Cells were centrifuged, the inoculum was removed, cells were washed three times with RPMI media, and cells were incubated at 37°C for 30 min to stimulate uptake. Cell were fixed with 4% formaldehyde and external bacteria were stained with an anti-LPS (green) antibody. Cells were subsequently permeabilized with 0.1% Triton-X 100 and stained with an anti-LPS (red) antibody to stain total bacteria and DAPI to stain host and bacterial DNA. The number of infected cells (red only) was determined and expressed as a percent of total cells. Data was tabulated from 20 fields in triplicate. Data are representative of 2 independent experiments. Statistical significance was determined using One-way ANOVA with Tukey as a post-test and yielded a significant difference of ^***^p < 0.001, ^**^p < 0.01, or ^*^p < 0.05.

Growth curve comparison and immunofluorescence microscopy of inclusions formed by serovars L2, D, and A in urogenital, conjunctival cells, HeLa cells, and PMA stimulated THP-1 cells revealed significant differences in intracellular replication and inclusion formation between the serovariants (Figures 1, 2). To determine if these differences could be due to defects in uptake, we compared uptake of L2, D, and A in each cell-type. No significant difference in serovar uptake was noted in HeLa cells, however reduced uptake of serovars D and A was noted compared to L2 in all other cell-types (Figure 3, Figure S2). Intriguingly, no difference in uptake was noted for serovar A compared to D. These results suggest that while differences in serovar-specific uptake may play a role in cell-specific chlamydial infection, uptake differences are not the sole factor that govern restriction of serovar A replication in urogenital cells as similar numbers of bacteria are taken up for serovar D which robustly replicates in these cells.

### The Host Cytokine Response to *C. trachomatis* Is Cell Type and Serovar Specific

To determine if the host innate immune response to chlamydial serovars differs and could potentially contribute to the differential ability of serovariants to grow in specific cell types, we infected HVEC, HCK, HFK-2, HCjE, HeLa, and THP-1 cells with serovars L2, D, and A and assessed the production of 36 cytokines using a human cytokine array. Notably, this array can detect the vast majority of cytokines that *Chlamydia* is known to induce upon infection. We compared cytokine profiles at 24 and 48 h post-infection, representing early and late stages of infection (Figure S3). Compared to the other cell-types, the HeLa cells were less immunoreactive, which is in line with previous observations indicating that they have many abnormalities in immune signaling pathways (Masters, 2002; Landry et al., 2013; Mittelman and Wilson, 2013) (Figure S1). In agreement with previous studies, we observed induction of Groα, IL-1α, IL-8, and IL-6 following infection of HeLa cells with serovar L2 (Rasmussen et al., 1997) (Table 1). To the best of our knowledge, these are the first data demonstrating cytokine production by *Chlamydia* infected vaginal epithelial cells, foreskin keratinocytes, and ectocervical keratinocytes, suggesting that they may play a major role in initiating the first immune mobilization early during chlamydial infection.

**Table 1:** Cytokine profiles of HeLa, HVEC, HCK, HFK-2, HCjE, or PMA stimulated THP-1 cells infected with L2, D, or A for 24 or 48 h.

	**HeLa (24/48 h)**			**HVEC (24/48 h)**			**HCK (24/48 h)**			**HFK-2 (24/48 h)**			**HCjE (24/48 h)**			**THP-1 (24/48 h)**		
	**L2**	**D**	**A**	**L2**	**D**	**A**	**L2**	**D**	**A**	**L2**	**D**	**A**	**L2**	**D**	**A**	**L2**	**D**	**A**
CD40 Ligand																–/NE	–/NE	2.6/NE
G-CSF													2.8/3	1.6/1.5	2.5/–			
GM-CSF				NE/7.9	NE/1.9	NE/1.8	–/4.9	–/1.8	–/2.4	NE/37.9	NE/NE	NE/NE	14.3/74.5	NE/NE	NE/NE			
CXCL1/GROα	15.7/37.4	NE/10.9	NE/7.2				–/2.0	–/–	–/2.4	NE/2.0	NE/–	NE/1.5	2.1/–	–/–	2.0/–	31.3/8.1	3.6/NE	4.7/NE
ICAM-1	–/1.6	1.5/–	–/–				–/–	–/–	–/1.9	–/1.8	–/–	1.6/1.5						
IL-1α	21.6/59.4	NE/23.7	NE/8.9				–/1.9	–/–	–/1.9	1.5/–	–/–	–/–				6.7/NE	NE/NE	3.4/NE
IL-1β				–/–	–/1.9	–/–	–/–	–/–	–/2.1	1.5/6.4	1.5/–	1.9/1.6	–/1.6	–/–	–/–	–/77.8	–/69.5	–/64.7
IL-1ra				–/–	–/–	–/1.5	–/–	1.6/1.7	–/1.6									
IL-6	NE/28.3	NE/NE	NE/NE	NE/2.0	NE/1.9	NE/1.8	–/2.4	–/2.8	–/3.7				19.2/6.9	NE/NE	NE/NE	3.7/NE	3.7/NE	3.7/NE
IL-8	9.2/91.8	7.1/16.1	7.1/NE							NE/30.3	NE/7.1	NE/9.9	2.0/2.1	–/–	1.6/–	3.4/22.4	2.5/18.1	3.2/13.2
IL-16																–/2.3	–/–	2.7/–
IL-18	82.2/–	51.6/–	24.9/–	–/2.6	–/–	–/–										–/6.9	–/–	–/–
IL-32α							NE/NE	NE/NE	4.9/NE				7/NE	NE/NE	NE/NE			
CXCL10/IP-10				NE/68.2	NE/–	NE/1.6							NE/9.9	NE/10	NE/1.6	52.2/81.8	4.0/NE	5.8/5.4
CXCL12/SDF-1													–/5	–/–	–/–			
MIF	–/–	1.5/–	1.6/1.5	–/1.6	–/–	–/2.0	–/–	1.5/1.7	–/–				–/–	–/1.9	–/1.5	–/–	–/–	1.9/–
MIP-1α/MIP-1β																–/12.4	–/11.3	–/10.1
CCL5/RANTES	NE/NE	NE/NE	NE/22.1	NE/NE	NE/NE	NE/15.3												
SerpinE1/PAI-1	–/–	2.1/–	2.7/–													1.7/NE	–/NE	–/NE

*Fold-change compared to uninfected cells is shown in blue (up) or red (down). NE, not expressed; –, no change. Only those with >1.5-fold change are shown*.

**Figure 4:**
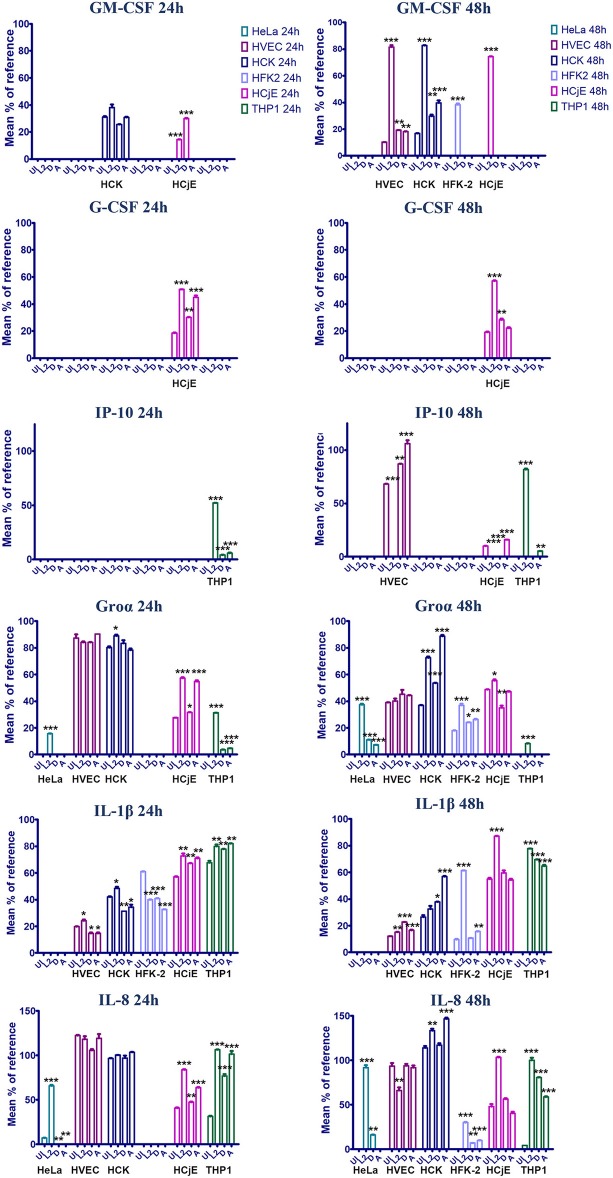
GM-CSF, G-CSF, Groα, IL-1β, IL-8 production in HVEC, HCK, HFK-2, and HCjE cells infected with *C. trachomatis* serovar L2, D, or A for 24 or 48 h. Cells were infected at an MOI of 1 with each serovar and at 24 or 48 h post-infection cytokine production was assessed using the human proteome profiler array. Signal was normalized to reference spot as described in the materials and method section and the signal percentage was expressed as a mean percent to the reference. Asterisks indicate significant differences between uninfected cells and infected cells. One-Way AVOVA was used to determine statistical significance (^*^P ≤ 0.05, ^**^P ≤ 0.01, ^***^P ≤ 0.001).

At 24 h post-infection, very few differences in cytokine production were noted. No difference in cytokine production was noted for HVECs at 24 h post-infection and only a mild induction of MIF and IL-1rα was observed in HCK cells infected with serovar D. Interestingly, a ~1.5–2-fold decrease in IL-1β production was noted in human foreskin keratinocytes at 24 h regardless of the serovar, whereas IL-β production was elevated in L2 and A infected cells at 48 h (Figure 4). This suggests that *C. trachomatis* may actively repress inflammasome activation in specific cell-types early during infection. Furthermore, HFK cells may naturally resist infection due to a heightened early inflammasome response. In contrast, infection of HCjE cells resulted in induction of G-CSF at 24 h for all serovars, but only L2 and A induced Groα and IL-8 production at this early time point (Figure 4).

At 48 h post-infection, we observed significant differences in cytokine production in *C. trachomatis* infected cells as well as several cytokines that were produced in a serovar- and cell-specific manner (Table 1, Figure 4). In line with previous observations, we noted that infection with *C. trachomatis* stimulates GM-CSF production (Rasmussen et al., 1997; Darville et al., 2001; Derbigny et al., 2010; Porcella et al., 2015; Lehr et al., 2018). Here we show that L2 induces a 4 or 2-fold increase in GM-CSF production compared to D or A infected HVEC or HCK cells, respectively. Intriguingly, we only observed GM-CSF production in HFK-2 (Shen et al., 2000) cells when infected with serovar L2 whereas no GM-CSF was produced in response to D or A infection at either time-point. Interestingly, we only observed G-CSF production in HCjE cells, and this occurred in a serovar-independent manner at 24 h. However, at 48 h only infection with L2 or D stimulated G-CSF production.

We observed an up-regulation of IL-6 production in HVEC or HCK cells regardless of the serovar, but no IL-6 production was noted in HFK-2 cells. Interestingly, IL-1β which we noted only in HFK-2 cells at 24 h post infection can suppress IL-6 dependent signaling Notably, we observed a 30.3- or 7.1-fold increase in IL-8 production for HFK-2 cells infected with L2 or D, respectively. We did not observe any significant difference in IL-8 levels for HVEC or HCK infected cells. These results suggest that keratinized male foreskin epithelial cells and female ectocervical cells in addition to vaginal epithelial cells mount unique responses to *C. trachomatis* infection.

In stark contrast to the cytokine profile of urogenital cells, infection of HCjE with either serovar L2 or A elicited production of several proinflammatory cytokines (G-CSF) and chemokines (IL-8, Groα) at 24 h. Notably, infection with serovar D only elicited production of G-CSF and intriguingly MIF and IP-10 were repressed (Table 1). Only infection with L2 stimulated production of GM-CSF, IL-1β, IL-6, IL-32, and CXCL12. Collectively our results show that infection of the conjunctival epithelial cells with L2 or A elicits the production of more proinflammatory cytokines and chemokines than infection with serovar D. It is intriguing to speculate that the induction of a more robust immune response may serve as a potential reason why infection with LGV isolates are especially virulent and are considered an ocular emergency whereas infection with genital isolates results in self-limiting conjunctivitis.

CXCL10/IP-10 is secreted by endothelial cells and fibroblasts in response to IFN-γ and serves as a chemoattractant for T-cells, macrophages, dendritic cells, and NK cells. Here we show that in both HVEC and HCjE cells infected with serovar L2, production of IP-10 is significantly repressed (Figure 4). Conversely, infection of either cell type with serovar A stimulates IP-10 production. Interestingly, serovar D represses IP-10 in the conjunctiva but in HVEC cells no significant difference in IP-10 levels were observed compared to uninfected cells.

In contrast to infection of epithelial cells, infection of PMA-stimulated THP-1 cells resulted in increased production of CXCL1/Gro α, IL-1α, and IL-8 at 24 h post-infection while IL-6 was repressed by all serovars at this timepoint. We also noted induction of IP-10 by all 3 serovars at 24 h. By 48 h, we observed up-regulation of IL-1β and IL-8 regardless of the serovar. Uniquely, serovar L2 induced expression of the neutrophil chemoattractant CXCL1/Gro α, but repressed IL-16 and IL-18. It is intriguing that the inflammasome associated cytokine IL-18 is differentially modulated by serovar L2 in macrophages and may mediate the differential survival of serovar L2 vs. D and A.

Since serovar D and A did not replicate or form inclusions in PMA-stimulated THP-1 cells (Figures 1, 2), we sought to determine whether bacterial protein synthesis is required for induction of cytokine production. As shown in Figure S4 inhibition of bacterial protein synthesis with chloramphenicol did not significantly alter host cytokine production in response to *C. trachomatis* infection. However, inhibition of serovar A protein synthesis did result in production of IL-6 and IL-8 suggesting serovar A may uniquely repress production of these two cytokines.

Collectively, the results of our cytokine arrays indicate that the host response is mounted in a cell-type and serovar-specific manner. Global comparison of the cytokine response in each cell-type based on the serovar (Table 2) revealed that infection with serovar L2 or A resulted in production of more proinflammatory cytokines and chemokines. In contrast, in many of the cell types tested D elicited a minimal response or repressed induction of proinflammatory cytokine and chemokine production.

**Table 2:** Comparison of proinflammatory cytokine, anti-inflammatory cytokine, and chemokine production based on serovar.

	**L2**	**D**	**A**
**Proinflammatory cytokines**			
G-CSF	HCjE (24, 48)	HCjE (24, 48)	HCjE (24)
GM-CSF	HVEC (48), HCK (48), HFK-2 (48), HCjE (48)	HVEC (48), HCK (48)	HVEC (48), HCK (48)
IL-1 alpha	HeLa (24, 48), HCK (48), HFK-2 (24), THP1 (24)	HeLa (48)	HeLa (48), HCK (48), THP1 (24)
IL-1 beta	HFK-2 (24, 48), HCjE (48), THP1 (48)	HVEC (48), HFK-2 (24), THP1 (48)	HCK (48), HFK-2 (24, 48), THP1 (48)
IL-6	HeLa (48), HVEC (48), HCK (48), HCjE (24, 48), THP1 (24)	HVEC (48), HCK (48), THP1 (24)	HVEC (48) HCK (48), THP1 (24)
IL-18	HeLa (24), HVEC (48), THP1 (48)	HeLa (24)	HeLa (24)
IL-32 alpha	HCjE (48)	HeLa (24)	HeLa (24, 48)
MIF	HVEC (48)	HCjE (48)	HVEC (48), HCjE (48)
**Anti-inflammatory**			
IL-1ra		HCK (24, 48)	HVEC (48), HCK (48)
**Chemokines**			
CCL5/RANTES			HeLa (48)
CXCL1/GRO alpha	HeLa (24, 48), HCK (48), HFK-2 (48), HCjE (24), THP-1 (24, 48)	HeLa (48)	HeLa (48), HCK (48), HFK-2 (48), HCjE (24)
IL-8 (CXCL8)	HeLa (24), HFK-2 (48), HCjE (24, 48)	HeLa (24), HFK-2 (48)	HeLa (24), HFK-2 (48), HCjE (24)
IP-10 (CXCL10)	HVEC (48), HCjE (48), THP1 (24, 48)	HCjE (48), THP1 (24)	HVEC (48), HCjE (48), THP1 (24, 48)
CXCL12/SDF-1	HCjE (48)		
IL-16	THP1 (48)		THP1 (24)
**Miscellaneous**			
CD40 Ligand			THP1 (24)
ICAM-1	HeLa (48), HFK-2 (48)	HeLa (24)	HCK (48), HFK-2 (24, 48)
MIP-1 alpha/MIP-1 beta	THP1 (48)	THP1 (48)	THP1 (48)

*Fold-change compared to uninfected cells is shown in blue (up) or red (down). Only those with >1.5-fold change are shown. Timepoints (24 or 48 h post-infection) are shown in parenthesis*.

## Discussion

While it is well-established that *C. trachomatis* serovars cause different diseases depending on the type of cell and tissue infected, how specific cell types respond early to serovar specific infection and how these serovars proliferate in cells that may be some of the first to encounter the pathogen early during infection is largely unknown. This significant gap in our understanding of chlamydial cellular tropism and the host response to *Chlamydia* can mostly be attributed to the fact that the majority of studies have focused on a single cell-type and serovar to evaluate chlamydial infection or have focused on cell-types that are more permissive to chlamydial proliferation. In the current study, we sought to characterize the early host and bacterial response to infection in specific cells, some of which are not canonically associated with ascending chlamydial infection, by representative trachoma and LGV serovariants. Using a combination of early passage immortalized urogenital and conjunctival epithelial cells, PMA stimulated THP-1 cells, and HeLa cells; we show significantly different proliferation rates and inclusion formation characteristics of *C. trachomatis* serovariants. Epithelial cells of the foreskin, vagina, and ectocervix represent cell-types that are likely exposed to large numbers of the pathogen early during attempted colonization and these cells may actually define whether *C. trachomatis* indeed succeeds in colonization and/or ascends to more permissive tissues of the body. Notably, we show for the first time that some *C. trachomatis* serovars can robustly replicate in vaginal epithelial and keratinized foreskin cells, suggesting that these cells may be the initial targets of chlamydial infection and the first sentinels to launch an immune response. Furthermore, we demonstrate that key cytokines such as GM-CSF, RANTES, GROα, IP-10, IL-8, IL-18, and IL-1β are differentially produced in response to specific serovars infecting different cell-types suggesting that serovars have evolved modes to suppress or even enhance cytokine production in different cell types.

*C. trachomatis* serovars exhibit significant differences in growth rate with ocular tropic isolates being very slow growing, genital strains possessing an intermediate growth rate, and LGV strains exhibiting the fastest growth rate (Miyairi et al., 2006). Early studies proposed that these observed differences in growth rate could be due to different lengths of time required to complete the developmental cycle or constraints implemented by the host cell (Miyairi et al., 2006). Prior studies demonstrated that the slow growth of ocular isolates could be alleviated through growth in conjunctival cells or by co-infection with serovar D or L2 (Miyairi et al., 2006). This suggests that specific aspects of host cell biology promote or restrict replication of *Chlamydia* in a biovar-specific manner. The fact that the slow growth phenotype associated with ocular isolates can be overcome by co-infection with other serovars may suggest that a secreted factor plays a role in cellular tropism. In line with previous observations (Miyairi et al., 2006; Hu et al., 2013), we show that urogenital, LGV, and trachoma isolates infect and replicate in conjunctiva epithelial cells. However, growth of serovar A in these cells more resembles the intermediate growth rate of serovar D. Conversely, we show that human vaginal epithelial cells, foreskin keratinocytes, and ectocervical keratinocytes restrict replication and inclusion development of the ocular tropic trachoma isolate, serovar A while promoting growth of L2 and D. Intriguingly, we observed an increase in inclusion size and total bacterial burden (~1-3 logs) when urogenital or LGV serovars were grown in these cell-types compared to HeLa cells. Collectively, our results indicate that early passage immortalized vaginal epithelial cells, urogenital keratinocytes, and conjunctiva cells may be appropriate *in vitro* models for studying early infection dynamics than Hela cells, which represent the most prolific model in the literature.

The ability of bacterial pathogens to establish a chronic infection and stably colonize the host requires evasion of the host immune response. During chlamydial infection of the genitourinary tract, macrophages and monocytes are recruited to the site of infection by following the chemoattractant gradient of cytokines and chemokines produced by infected epithelial cells (Darville and Hiltke, 2010; Lausen et al., 2018). Efficient phagocytosis and killing of bacteria by macrophages and other phagocytes are necessary to prevent the spread and dissemination of chlamydiae. Studies evaluating the ability of *Chlamydia* to infect, replicate, and survive in macrophages has produced conflicting results, mostly due to the use of various serovars and species as well as different infection readouts (Sun et al., 2012; Al-zeer et al., 2013; Datta et al., 2014; Lausen et al., 2018). Here we compared intracellular replication and inclusion development of serovars L2, D, and A in PMA stimulated THP-1 cells. While reduced compared to results obtained in epithelial cells, L2 is able to infect and replicate in macrophages. Importantly, regrowth assays on HeLa cells (Figure 1) resulted in the formation of mature inclusions, indicating the bacteria remain viable. Growth of both serovar D and A was severely impaired in these conditions and re-infection on HeLa monolayers produced few inclusions, indicating most of the bacteria were not viable or had been irreparably damaged within the stimulated macrophage. These findings are supported by our immunofluorescence microscopy analysis in which only individual bacteria are observed at 48 h post-infection. LGV isolates are capable of trafficking to the regional draining lymph node where they cause a systemic disease, the underlying mechanisms of which are poorly understood. We speculate that the ability of LGV, but not genitourinary or trachoma isolates, to survive in macrophages may promote bacterial dissemination. Furthermore, our results suggest that L2 may trigger secretion of chemotactic factors that enhance macrophage recruitment to the site of infection as we discuss below. Collectively these results suggest that LGV isolates have evolved sophisticated methods to avoid degradation in macrophages, however future work is needed to elucidate the mechanistic underpinnings of how LGV survives in macrophages.

Interferon (IFN)-γ-inducible protein 10 (IP-10), also known as C-X-C motif chemokine 10 (CXCL10), is secreted by leukocytes, neutrophils, epithelial, and endothelial cells in response to IFN (Liu et al., 2011). IP-10 activates the CXCR3 receptor expressed on activated T-cells, dendritic cells, macrophages, and natural killer cells; which induces chemotaxis (Liu et al., 2011). Previous studies using *C. muridarum* demonstrated that IP-10 expression was up-regulated early following genital infection in mice and expression was maintained during productive infection (Belay et al., 2002). Subsequent studies evaluating the cell-types and IFN response that is required to elicit IP-10 production during *C. muridarum* infection of the genital tract revealed that mouse macrophages, mouse embryonic fibroblasts (MEFs), and lung fibroblasts produce IP-10 and IFN-β and this is dependent on bacterial growth (Nagarajan et al., 2005). In line with previous observations, we show here that IP-10 is produced by PMA stimulated THP-1 cells at 24h post-infection regardless of the serovar. Intriguingly, we observed a 13- or 9-fold increase in IP-10 production in L2 infected cells compared to those infected with serovar D or A, respectively (Figure 4, Table 1). Differences in the levels of IP-10 induction by serovar L2 may suggest that active bacterial replication enhances IP-10 production as we observe minimal increases in bacterial burden in THP-1 cells infected with serovar D or A (Figure 1). Furthermore, IP-10 levels remain constant at 48h for these two serovars but were elevated at 48 h for L2 infected cells. Uniquely, we show here that L2 represses IP-10 in HVEC and HCjE cells whereas serovar A induces IP-10 expression in these cells. Recent work has also demonstrated an increase in IP-10 production in endometrial stromal fibroblast infected with ocular isolates (A or Ba) compared to genital isolates (D or E) (Jolly et al., 2019). Collectively, these results suggest that IP-10 induction occurs in a cell-type and serovar-specific manner and infection of macrophages during human infection may be important for inducing T cell chemotaxis during *C. trachomatis* infection, which may promote tissue pathology. Intriguingly, we did not detect IFNγ in any of our cell-types and IFNβ, which was previously shown to be required for IP-10 induction (Nagarajan et al., 2005), is not included in the human proteome array. It will be interesting to evaluate the contribution of IFNβ to *C. trachomatis* serovariant infection in the future.

Granulocyte-macrophage colony stimulating factor (GM-CSF) is a cytokine produced by macrophages, endothelial cells, fibroblasts, and T cells that stimulates the production of granulocytes and monocytes (Shi et al., 2006). GM-CSF binds to receptors expressed on endothelial cells, monocytes, macrophages, lymphocytes, and granulocytes resulting in autophosphorylation of JAK2 and subsequent activation of STAT5 and MAPK (Shi et al., 2006). GM-CSF has been observed in genital secretions of mice infected with *C. muridarum* (Darville et al., 2001) and is also induced in HeLa cells infected with *C. trachomatis* serovar L2 (Rasmussen et al., 1997). Here we show that infection of multiple cell-types by serovar L2 stimulates GM-CSF production (Figure 4, Table 1). While we observed GM-CSF in HVEC and HCK cells infected with serovars D or A, a 4-fold or 2-fold increase in GM-CSF production in HVEC or HCK cells was noted when infected with serovar L2. Intriguingly, we only observed GM-CSF production in HCjE or HFK-2 cells when infected with serovar L2. Production of GM-CSF by epithelial cells stimulates production of monocytes which migrate into tissues and differentiate into macrophages. It is interesting that serovar L2, which is capable of replicating in macrophages, stimulates elevated levels of GM-CSF compared to other *C. trachomatis* serovars that cannot survive in macrophages. We speculate that *C. trachomatis* L2 has evolved a strategy to increase infiltration of macrophages to the infection site which may in turn be used as a Trojan horse mechanism for it to gain access to the lymph system. Whether GM-CSF production and macrophage infection is necessary for serovar L2 to cause systemic disease warrants further study.

Infection with *C. trachomatis* involves a complex interplay between many cell-types that orchestrate cytokine and chemokine production to stimulate leukocyte, granulocyte, and monocyte production and homing to the infection site. Our study and recent published work (Jolly et al., 2019) suggest that stromal fibroblasts and genitourinary epithelial cells play an important role in the initial response to *C. trachomatis*. Importantly, we and others (Jolly et al., 2019) have shown that the innate immune response to *C. trachomatis* is mounted in a cell-type and serovar-specific manner. We show for the first time that human foreskin keratinocytes can be infected by *C. trachomatis* and that serovars D and L2 form normal inclusions and robustly replicate in these cells suggesting that the human foreskin may be infected during early stages of colonization. Furthermore, HFK-2 cells responded to infection by secreting a plethora of pro-inflammatory cytokines that may play a key role in limiting infection in these cells following exposure and may drive ascension of *C. trachomatis* to more sensitive tissues of the urethra and epididymis. This study suggests that ectocervical keratinocytes and non-keratinized vaginal epithelial cells may play an integral role in initiating the early innate response to *Chlamydia* infection and provide a major barrier to infection of more permissive tissues. Furthermore, this study has introduced several cell lines that represent some of the first cell-types likely to encounter and respond to *C. trachomatis* making them more relevant models than HeLa or Hep2 cells for the study of the host immune response to *Chlamydia* infection.

## Data Availability Statement

All datasets generated for this study are included in the article/Supplementary Material.

## Author Contributions

RF, SA, AM, FG, and MW conducted the experiments. RF, AK, and MW conceived the experiments. RF and MW wrote the manuscript.

### Conflict of Interest

The authors declare that the research was conducted in the absence of any commercial or financial relationships that could be construed as a potential conflict of interest.
