# Supplementary material for: Chlamydia trachomatis Serovars Drive Differential Production of Proinflammatory Cytokines and Chemokines Depending on the Type of Cell Infected
Source: Front Cell Infect Microbiol. 2019 Nov 26;9:399. doi: 10.3389/fcimb.2019.00399 (PMC6988789; doi:10.3389/fcimb.2019.00399)
Supplement: Supplementary file 1 [file Data_Sheet_1.docx]

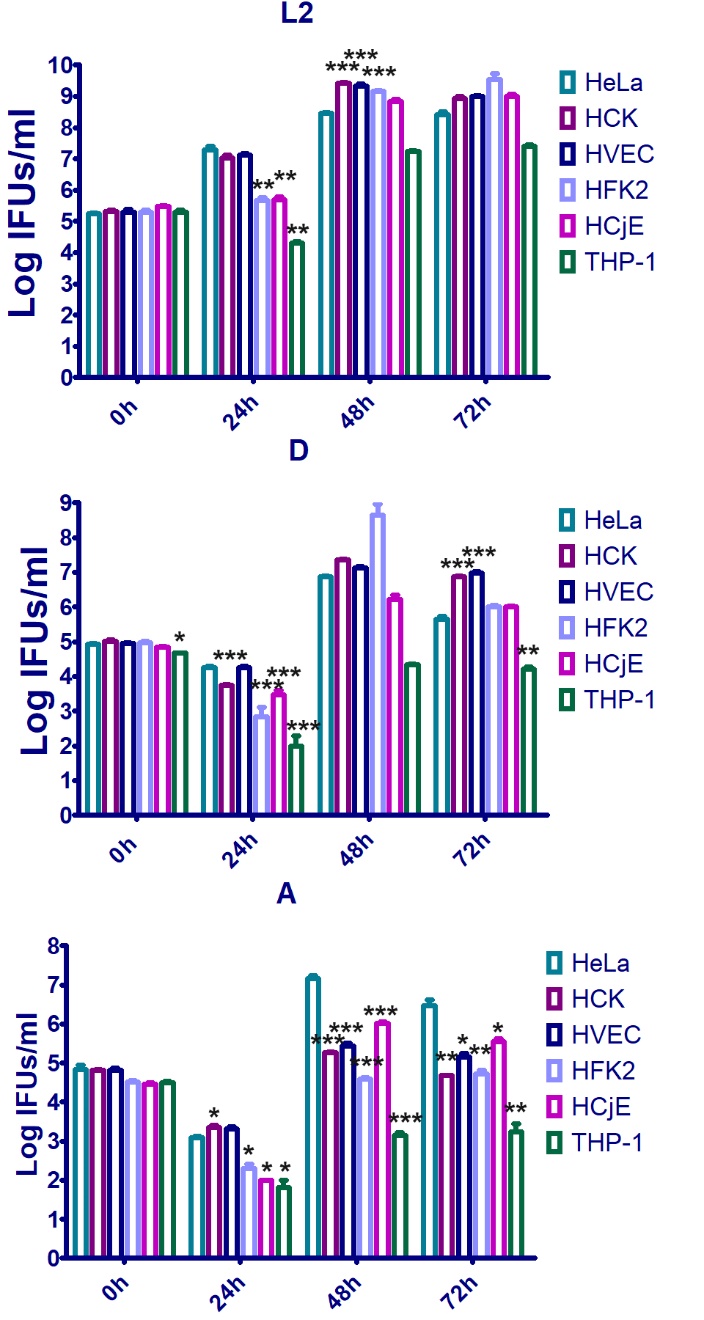


Fig. S1 Statistical analysis of trachoma, urogenital, or LGV isolates in early passage immortalized urogenital cells, conjunctiva cells, PMA stimulated THP-1 cells, and HeLa cells. Human vaginal epithelial cells (HVEC), human foreskin keratinocytes (HFK-2), human ectocervical (HCK), human conjunctival epithelial cells (HCjE), PMA stimulated THP-1 cells, or HeLa cells were infected at a MOI of 1 with *C. trachomatis* 434/Bu (L2), D/UW-3/CX (D), and A/HAR-13 (A). At 0, 24, 48, or 72h post-infection, host cells were lysed in water and lysates were plated on fresh HeLa monolayers to enumerate the number of infectious forming units (IFUs). Data are representative of 3 independent experiments. One-Way AVOVA with Tukey as a post-test was used to determine significance compared to HeLa cells. P < 0.05 = *, P < 0.01 = **, P < 0.001 = ***.


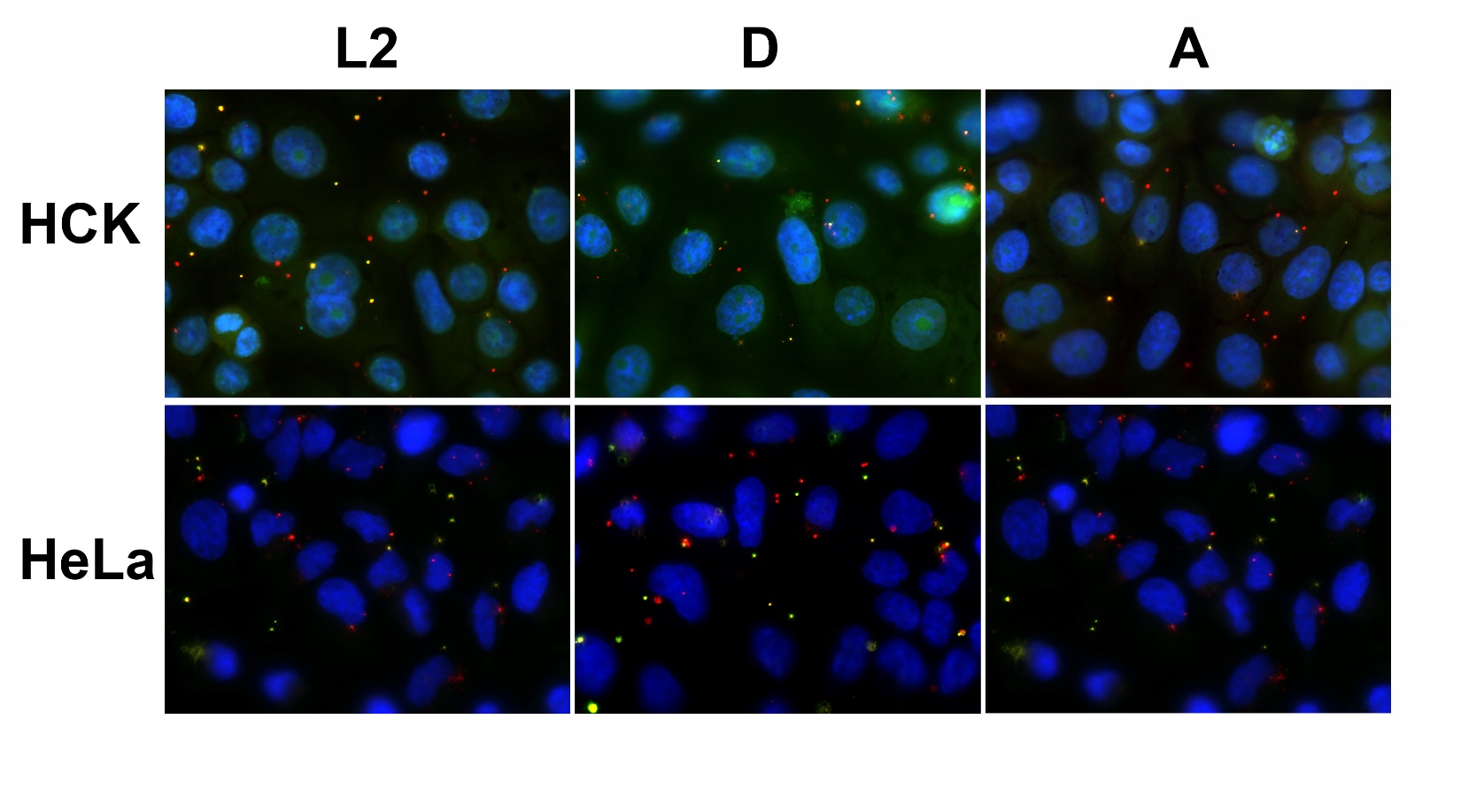


Fig. S2 Representative images of differential uptake of *C. trachomatis* serovars by HCK and HeLa cells. Cells were infected at a MOI of 1 with *C. trachomatis* 434/Bu (L2), D/UW-3/CX (D), and A/HAR-13 (A). Cells were centrifuged, the inoculum was removed, cells were washed three times with RPMI media, and cells were incubated at 37°C for 30min to stimulate uptake. Cell were fixed with 4% formaldehyde and external bacteria were stained with an anti-LPS (green) antibody. Cells were subsequently permeabilized with 0.1% Triton-X 100 and stained with an anti-LPS (red) antibody to stain total bacteria and DAPI to stain host and bacterial DNA. The number of infected cells (red only) was determined and expressed as a percent of total cells. Data was tabulated from 20 fields in triplicate. Data are representative of 2 independent experiments. Statistical significance was determined using One-way ANOVA with Tukey as a post-test and yielded a significant difference of p<0.001 (***), p<0.01(**), or p<0.05 (*).

Fig. S3 Cytokine profiles of HeLa, HVEC, HCK, HFK-2, HCjE, and PMA stimulated THP-1 cells infected with *C. trachomatis* serovar L2, D, or A for 24 or 48h. Cells were infected at an MOI of 1 with each serovar and at 24 or 48h post-infection cytokine production was assessed using the human proteome profiler array. Signal was normalized to reference spot as described in the materials and method section and the signal percentage was expressed as a mean percent to the reference.

Fig. S4 Cytokine profiles of PMA stimulated THP-1 cells infected with *C. trachomatis* serovar L2, D, or A for 48h and treated with chloramphenicol (CAM). Cells were infected at a MOI of 1 with each serovar and at 48h post-infection cytokine production was assessed using the human proteome profiler array. Signal was normalized to reference spot as described in the materials and method section and the signal percentage was expressed as a mean percent to the reference.
